# Cell-specific multi-target mechanisms of cotinine in cervical cancer oncogenesis and progression

**DOI:** 10.1007/s12672-026-05375-5

**Published:** 2026-06-12

**Authors:** Jiaming Cheng, Yiyang Shi, Qianqian Jiang, Changsong Lin

**Affiliations:** 1 Department of Bioinformatics, Nanjing Medical University, Nanjing, 211166 Jiangsu China; 2 Nanjing University of Chinese Medicine, Nanjing, 210023 Jiangsu China

**Keywords:** Cotinine, Epithelial cells, Transformed cells, Cervical cancer, Network toxicology

## Abstract

**Background:**

Cervical cancer is the fourth most common malignant tumor in women globally, with tobacco smoke being a major environmental risk factor. The tobacco-specific metabolite cotinine may promote cancer progression through mechanisms such as chronic inflammation and genomic instability, but its precise molecular targets and interactions remain unclear.

**Methods:**

We integrated network toxicology and multi-omics for target prediction, validated by molecular biology experiments and dynamic simulations. The workflow included database screening of cotinine-related targets, identification of key modules via single-cell analysis and hdWGCNA, integration of differential gene analysis to obtain potential targets, determination of core targets using protein-protein interaction networks, pathway enrichment and machine learning, transcriptomic profiling of cotinine-induced cell type-specific pathway regulation in H8 and HeLa cells, and further validation through molecular docking, molecular dynamics simulations, and in silico knockout.

**Results:**

Initial screening identified 151 cotinine-related targets, refined to 17 key candidates via integrated multi-omics analysis and machine learning. Molecular studies revealed multi-level carcinogenic mechanisms: in H8 cells, cotinine promoted tumorigenesis via chronic inflammation, differentiation defects, ECM remodeling, and signaling dysregulation; in HeLa cells, it accelerated progression/metastasis through dysregulated proliferation, matrix degradation, impaired metal ion transport, and cytokine storms. Validated targets—*KAT2B* (in H8 cells) and *CA9*, *CCNB1*, *LAGE3* (in HeLa cells)—were significantly downregulated in cervical tumors. Molecular docking and dynamics simulations confirmed stable cotinine-target binding.

**Conclusion:**

This study systematically demonstrates that cotinine exerts its carcinogenic effects by targeting *KAT2B*, *CA9*, *CCNB1*, and *LAGE3*, thereby dysregulating multiple signaling pathways to induce tumorigenesis in epithelial cells and accelerate progression/metastasis in malignant cells. Elucidation of this multi-target toxicity mechanism provides a critical theoretical foundation for developing targeted detoxification strategies against tobacco-associated cervical cancer.

**Graphical Abstract:**

**Figure:**
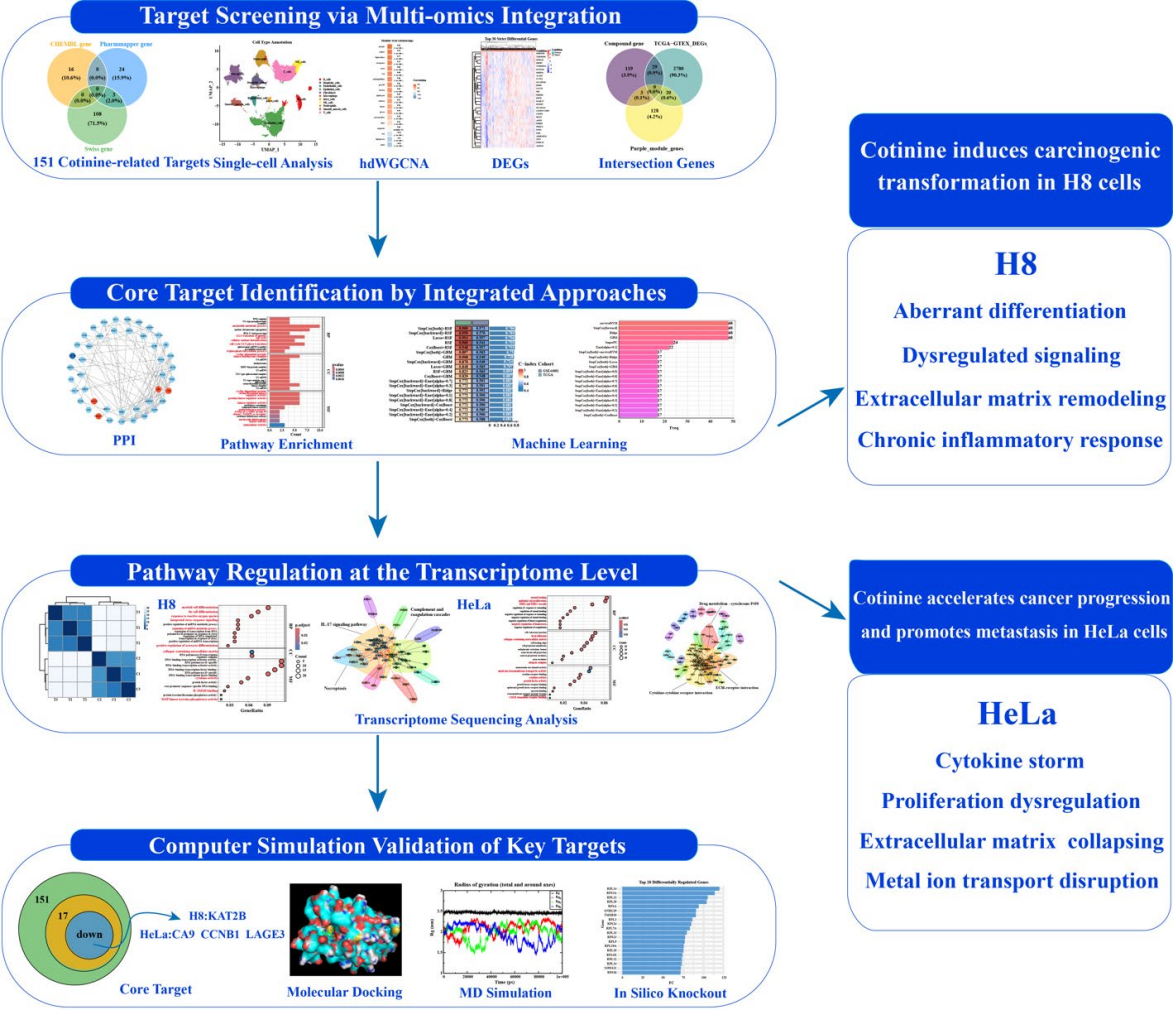

**Supplementary Information:**

The online version contains supplementary material available at 10.1007/s12672-026-05375-5.

## Highlights

- Integrating network toxicology with multi-omics analysis for computational prediction.
- Establishing a robust bidirectional validation framework integrating molecular biology experiments with computational approaches.
- Elucidating distinct molecular targets and differential pathological impacts of cotinine on both epithelial cells and malignant cells during cervical cancer progression.

**Supplementary Information:**

The online version contains supplementary material available at 10.1007/s12672-026-05375-5.

## Introduction

Cervical cancer (CC) is the fourth most common malignancy and leading cause of cancer death in women globally. While developed nations show declining rates due to improved screening and lifestyle changes, low-income countries face rising incidence and mortality [1]. The pathogenesis of cervical cancer involves multifactorial interactions, with tobacco smoking, high parity, and prolonged oral contraceptive use serving as significant cofactors in disease progression [2]. These risk factors exert distinct pathophysiological effects on the cervical microenvironment through different biological pathways. Notably, smoking demonstrates a strong dose-dependent association with cervical cancer risk, independent of HPV infection status [3]. Tobacco smoke exposure disrupts the oxidant-antioxidant balance in cervical tissue, leading to localized immunosuppression and elevated oxidative stress that create favorable conditions for cervical carcinogenesis [4]. However, current domestic and international research has principally demonstrated the cervix-specific carcinogenicity of tobacco smoking through epidemiological evidence, with limited systematic investigation into the underlying molecular mechanisms [5].

Current understanding of cervical carcinogenesis at the molecular level has reached relative maturity. The disease progression is recognized to be co-regulated by three fundamental mechanisms: genomic instability, epigenomic dysregulation, and hyperactivation of oncogenic signaling pathways [6]. These molecular alterations form an intricate network that drives malignant phenotypes including uncontrolled proliferation, apoptosis resistance, and enhanced metastatic potential. Tobacco-derived carcinogens directly interact with DNA to form adducts, inducing characteristic base substitutions that establish clear genotype-environment correlations during carcinogenesis. Notably, metabolized tobacco carcinogens form DNA adducts that accumulate in tissues in a dose-dependent manner with smoking intensity. These adducts induce endogenous methylation-associated CpG dinucleotide mutations, particularly characteristic G→T transversions that serve as molecular fingerprints of tobacco exposure [7].

Cotinine, the primary metabolite of nicotine, serves as a well-established biomarker for assessing smoking exposure [8]. Following metabolic activation, it generates highly reactive intermediates capable of inducing base mutations and chromosomal instability through DNA damage, while simultaneously reducing DNA repair protein activity and promoting oxidative DNA breaks [9, 10]. Notably, any nicotine exposure inevitably elevates leukocyte counts and proinflammatory cytokines, sustaining chronic inflammation in cervical tissues [11]. These molecular events collectively contribute to genomic instability accumulation, potentially inducing functional abnormalities in tumor-related genes and creating a microenvironment conducive to carcinogenesis [12].

Network toxicology integrates advanced bioinformatics models, algorithms, and database resources to construct a multidimensional “toxicant-active compound-toxicity-target-disease” network model at the experimental design stage [13]. Single-cell multi-omics technologies have revolutionized our ability to identify cellular heterogeneity and relationships, enabling systematic deconstruction of cancer-specific characteristics, thereby facilitating therapeutic target gene identification [14]. Machine learning provides sophisticated predictive models to narrow down potential targets associated with smoking-induced non-communicable diseases [15]. High-throughput RNA sequencing (RNA-Seq) technology enables high-resolution transcriptome profiling with precise gene expression quantification [16]. The resulting whole-transcriptome experimental evidence facilitates cross-validation for analytically predicted targets and computationally verified molecular docking results, ultimately establishing a complete research cycle from bioinformatic prediction to molecular biological verification. Molecular docking and dynamics simulations can predict interactions between tobacco components and key cellular proteins, thereby elucidating their metabolic impacts [17]. In silico knockout analysis is a highly efficient and systematic tool for investigating gene functions, and is particularly suitable for research scenarios where practical knockout (KO) experiments are unfeasible. It aids in further validating the critical regulatory roles of key target downregulation in cervical cancer progression.

In the present study, we aimed to systematically elucidate the cell-type-specific molecular mechanisms by which cotinine promotes cervical cancer initiation in normal cervical epithelial cells (H8) and accelerates progression and metastasis in transformed malignant cells (HeLa). Recognizing that the toxicological effects of tobacco metabolites are likely mechanistically distinct in cells at different stages of malignant transformation—a distinction largely overlooked in previous studies—we deployed an innovative triangular systems toxicology framework integrating network toxicology, single-cell multi-omics, machine learning-based target prioritization, transcriptomic profiling, molecular docking and dynamics simulations, and in silico knockout analysis to identify and validate cell-type-specific core molecular targets of cotinine in H8 versus HeLa cells, to delineate the distinct carcinogenic cascades triggered by cotinine in these two cellular contexts at the transcriptomic level, and to establish a bidirectional validation framework that triangulates computational predictions with molecular biological evidence. The broader significance of this work lies in its potential to inform the development of cell-type-specific biomarkers for risk stratification in tobacco-exposed female populations, the structure-guided design of cotinine antagonists for targeted chemoprevention, and evidence-based public health policies prioritizing smoking cessation in cervical cancer prevention. By distinguishing the differential pathological impacts of cotinine on epithelial cells versus malignant cells, this study provides a critical theoretical foundation that transcends the traditional single-cell-type analytical paradigm and opens new avenues for stage-specific therapeutic strategies against tobacco-associated cervical cancer.

## Materials and methods

### Acquisition of chemical components and targets of cotinine

Cotinine was characterized via multi-source database integration. Its physicochemical properties and biological parameters were systematically retrieved from PubMed, while canonical 2D structural descriptors (SMILES: CN1[C@@H](CCC1=O)C2=CN=CC=C2) were extracted from the PubChem database. Target prediction was performed using a tripartite strategy: ChEMBL Database for ligand–receptor interaction profiling; SwissTargetPrediction for chemical genomics-based prediction; and PharmMapper for 3D pharmacophore matching. All predicted targets were restricted to the *Homo sapiens* proteome.

### Raw data processing

The raw data of GSE168652, GSE197461 and GSE208653 for analysis were downloaded from the GEO online database. GSE168652 includes cervical cancer tissue and matched normal adjacent tissue from one patient with cervical cancer (2 samples total, platform GPL24676 Illumina NovaSeq 6000). GSE197461 comprises scRNA-seq data from 8 cervical cancer samples, including 3 HPV-positive squamous cell carcinoma (SCC) samples, 3 HPV-associated adenocarcinoma (HPVA ADC) samples, and 2 non-HPV-associated adenocarcinoma (NHPVA ADC) samples (platform GPL24676). GSE208653 contains scRNA-seq data spanning the full spectrum of cervical carcinogenesis, including normal cervix, HPV-infected normal cervix, high-grade squamous intraepithelial lesion (HSIL), and cervical cancer, with 2–3 replicates per stage (9 samples total, platform GPL24676). The Seurat package of R software was used to process the data. For quality control, cells were retained if they expressed at least 200 detected genes and had fewer than 15% mitochondrial gene content, as elevated mitochondrial content indicates damaged or dying cells; genes were retained if detected in at least 5% of cells within each cell type. Doublets were identified and removed using DoubletFinder. After filtering cells according to the above criteria, batch correction was performed using the Harmony package, and cell types were annotated using the SingleR software combined with manual annotation. Finally, the AUCell package was used to calculate the gene set scores.

### Single-cell analysis

We employed a single-cell RNA sequencing analytical pipeline to computationally characterize the expression patterns of 151 network toxicology-predicted genes across distinct cellular subpopulations. Briefly, spatially heterogeneous cell clusters were identified via UMAP dimensionality reduction. Subsequently, cell-type-specific marker gene expression profiles were established based on predefined thresholds, followed by quantitative assessment of target gene expression levels according to cellular classification results. Finally, the computational results were projected onto two-dimensional UMAP plots and visualized using violin plots. The mapping of the 151 cotinine-related targets to the scRNA-seq dataset was performed as follows: first, gene symbols of the 151 predicted targets were standardized; second, these gene symbols were cross-referenced with the gene expression matrix of the integrated scRNA-seq object, retaining only those targets present in the expression matrix; finally, for each cell, the expression values of all matched cotinine-related target genes were extracted for downstream AUCell-based gene set scoring, as described in Sect. 2.2.

### High-dimensional weighted gene co-expression network analysis with multi-omics functional characterization and network visualization of key modules

Scale-free co-expression network analysis was performed using the WGCNA package [18]. First, the optimal soft-thresholding power was determined via the “pickSoftThreshold” function in the WGCNA package combined with dynamic tree cutting. The selected power was β = 9. Next, the pairwise correlation matrix was converted into an adjacency matrix to establish scale-free network characteristics. Subsequently, the adjacency matrix was transformed into a Topological Overlap Matrix (TOM), and hierarchical clustering analysis was conducted based on the corresponding dissimilarity measures. Finally, module-trait associations were evaluated by analyzing the correlations between modules and samples to identify key modules. Given the strong correlation between the purple module and the target phenotype, this module was selected as the focus of subsequent investigations.

In addition to its significant phenotypic correlation, which is officially recommended by WGCNA for module-trait association analysis, the purple module demonstrated several lines of evidence supporting its selection as the key module. First, correlation matrix analysis revealed that the purple module exhibited strong positive correlations with multiple other key modules (including yellow, turquoise and lightyellow), with correlation coefficients approaching 1.0, positioning it as a highly connected “hub module” within the global co-expression network, suggesting that genes within this module coordinate with multiple biological processes rather than functioning in isolated pathways. Second, visualization of the intra-modular gene-gene interaction network showed exceptionally dense connections among purple module genes, indicating strong functional coherence and co-expression patterns. Collectively, these multi-dimensional criteria—high phenotypic correlation, strong inter-modular connectivity, and dense intra-modular cohesion—provide a robust rationale for prioritizing the purple module for downstream analysis.

Subsequently, the prominent features of the purple module were visualized, and Pearson correlation analysis was performed between the eigengene of the purple module and those of other modules to verify the biological rationality of selecting the purple module for in-depth research. Module-trait association analysis was further conducted, and bubble plots were generated to highlight the enrichment patterns of the purple module; additionally, the expression profiles of genes within the purple module across different subgroups were examined. Finally, module interaction networks were constructed to visualize the composition of module genes and their interrelationships. To emphasize the gene distribution patterns within the target purple module, a detailed visualization of its gene-gene interaction network was generated.

### Identification of DEGs in CC

Differential expression analysis was performed using the “limma” package to identify differentially expressed genes (DEGs) between disease samples and healthy controls, with the following thresholds: adjusted p-value (padj) < 0.01 and absolute log2 fold change (|logFC|) > 1.5 [19]. The “EnhancedVolcano” package was used to generate volcano plots for visualizing DEGs, with significant genes highlighted. Additionally, the “pheatmap” package was employed to construct expression heatmaps.

### Identification of key targets and generation of protein–protein interaction networks

We analyzed the overlap among differentially expressed genes (DEGs), database-predicted target genes, and hdWGCNA module genes, identifying 52 consensus genes at their intersection. To investigate the protein-protein interactions (PPIs) between these potential targets and known cervical cancer-associated genes, we performed comprehensive PPI analysis using the STRING database. In the constructed PPI network, nodes represent candidate proteins and edges represent their interaction relationships. To facilitate intuitive visualization and in-depth network analysis, we further refined the PPI network using Cytoscape software.

### Functional enrichment analysis

Functional enrichment analysis of these drug targets was conducted using the “clusterProfiler” package in R, incorporating both Gene Ontology (GO) and Kyoto Encyclopedia of Genes and Genomes (KEGG) analyses [20, 21]. The GO analysis classified these drug targets into three core ontologies: Cellular Component (CC), Biological Process (BP), and Molecular Function (MF), enabling systematic investigation of their intrinsic biological properties. For KEGG pathway enrichment, a stringent threshold of *p* < 0.01 was applied to screen for potential functionally relevant signaling pathways. Collectively, these complementary enrichment strategies comprehensively delineated the mechanistic involvement of cotinine in cervical carcinogenesis.

### Machine learning-based core gene screening

To systematically identify cotinine-associated diagnostic biomarkers for cervical cancer, we developed a comprehensive integrated machine learning framework that incorporates eleven classical algorithms—including Least Absolute Shrinkage and Selection Operator (Lasso), Support Vector Machine (SVM), Random Forest (RF), Generalized Linear Model Boosting (glmBoost), Stepwise Generalized Linear Model (Stepglm), Ridge regression, Elastic Net (Enet), Gradient Boosting Machine (GBM), Linear Discriminant Analysis (LDA), eXtreme Gradient Boosting (XGBoost), and Naive Bayes. Using transcriptomic data from the training set, this framework was employed to construct 150 predictive models. To ensure robust model performance, 5-fold cross-validation was implemented for hyperparameter optimization, and stratified sampling was adopted for the partitioning of training and internal validation sets. Model performance was rigorously assessed based on three key metrics: Area Under the ROC Curve (AUC), accuracy, and F1-score. Subsequently, the top-performing individual models were integrated via stacked ensemble learning to leverage complementary predictive capabilities. High-confidence models (AUC > 0.7) were selected, and candidate core genes were prioritized according to their feature occurrence frequency across these validated models. Finally, the utilization patterns of the models were visualized using the ggplot2 package, providing intuitive evidence to further validate the effectiveness and reliability of the identified candidate core genes.

### Cell source and culture

H8 and HeLa cell lines were purchased from Shanghai Fuheng Biotechnology Co., Ltd., where H8 cells are immortalized cervical epithelial cells. For routine maintenance, both cell types were cultured in Gibco DMEM basic (1×) complete medium supplemented with 10% fetal bovine serum (FBS), under standardized incubator conditions of 37 ℃, 5% CO₂, and saturated humidity. For cotinine treatment experiments, H8 cells in the logarithmic growth phase were first plated into T75 cell culture flasks at an optimal density. Following 24 h of conventional incubation to allow cell adhesion and stable proliferation, the culture medium was aspirated and replaced with fresh complete medium containing cotinine (HY-B1178, MCE) at varying concentrations. The treatment group was exposed to cotinine at a final concentration of 10 µM, whereas the control group was given an equal volume of complete medium to ensure consistent culture conditions. Both groups were further incubated for 24 h prior to the initiation of subsequent experimental procedures. HeLa cells were handled using the same culture and treatment procedures as H8 cells, with the only modification being the final concentration of cotinine in the treatment group, which was adjusted to 12.5 µM. All other parameters, including culture conditions, drug treatment duration, and control group settings, were identical to those used for H8 cells.

### Transcriptome sequencing analysis

Following rigorous quality control, adapter trimming, and quality filtering of raw FASTQ data, we established four sample groups—H8 treatment group, H8 control group, HeLa treatment group, and HeLa control group—each with three biological replicates to ensure experimental reproducibility. Differential gene expression analysis was performed using the DESeq2 package, a gold-standard tool for RNA-seq differential expression profiling, to identify significantly differentially expressed genes (DEGs). The resulting DEGs were visualized via volcano plots (to illustrate fold change and statistical significance) and heatmaps (to depict expression clustering patterns across samples). Upregulated genes meeting the stringent criteria of log2 fold change (log2FC) > 1 and adjusted P-value (padj) < 0.05 were subsequently selected for functional annotation. Utilizing the clusterProfiler R package, we conducted separate Gene Ontology (GO) enrichment analysis and Kyoto Encyclopedia of Genes and Genomes (KEGG) pathway annotation for the upregulated DEGs in H8 and HeLa cells, respectively. Only significantly enriched biological processes and signaling pathways (*P* < 0.05) were retained for downstream analysis to eliminate spurious associations. Specifically for H8 cells, Gene Set Enrichment Analysis (GSEA) was performed to delineate global expression patterns of pre-defined functional gene sets, complementing the individual gene-level enrichment results. Finally, transcription factor (TF) enrichment analysis was carried out to identify transcriptionally active TFs under distinct experimental conditions. This was followed by the construction of protein-protein interaction (PPI) networks, which were used to intuitively visualize the regulatory relationships between these candidate TFs and their target genes.

### Selection of key target

Following transcriptome profiling, we further refined the 17 key machine learning-predicted targets by imposing differential expression thresholds. For H8 cells, genes were filtered with a log2FC < −0.5 and adjusted p-value (p-adj) < 0.05, while for HeLa cells, a more stringent threshold of log2FC < −1 and p-adj < 0.05 was applied.

### Molecular docking and molecular dynamics simulation

To validate the interactions between cotinine and the identified core genes, we performed molecular docking simulations. First, the SDF-format structure of the cotinine ligand was retrieved from the PubChem database and converted to mol2 format using ChemOffice. Three-dimensional structures of the core target proteins were obtained from the Protein Data Bank (PDB) [22]. Using PyMOL (v 3.1.6.1), water molecules and bound ligands were removed from the target proteins, followed by structural preprocessing in AutoDock Tools (v 1.6), including hydrogenation, charge assignment, and definition of the docking root. Concurrently, the ligand geometry was optimized using the MMFF force field [23]. The docking grid was centered on the predicted active site, with dimensions adjusted according to ligand size and predicted binding modes. Semi-flexible docking between cotinine and each target protein was performed using AutoDock Vina (v 1.1.2), and the optimal binding conformations were selected based on binding energy scores. Results were analyzed and visualized using PyMOL.

Molecular dynamics (MD) simulations were performed using the GROMACS 2020 package with the AMBER99SB-ILDN force field. The protein-ligand complex was solvated in a dodecahedral box with TIP3P water molecules. Energy minimization, NVT, and NPT equilibration were sequentially performed. Finally, a 100-ns MD simulation was conducted. After removing artifacts from periodic boundary conditions, key parameters including root-mean-square deviation (RMSD) were calculated for subsequent analysis [24].

### In silico knockout analysis

To further validate the critical regulatory roles of *KAT2B* downregulation in H8 cells and *LAGE3* downregulation in HeLa cells during cervical cancer progression, we performed in silico knockout analyses of *KAT2B* and *LAGE3* using scTenifoldKnk—a computational knockout tool—based on our previously generated single-cell RNA sequencing (scRNA-seq) data, to infer their downstream functional impacts [25]. Briefly, we first constructed a gene regulatory network (GRN) that captures intergenic regulatory relationships using the scTenifoldKnk pipeline. Within this network, we mimicked the knockout of *KAT2B* and *LAGE3* by approximating the deletion of their corresponding nodes and performed unsupervised in silico perturbation assays. Using this strategy, we identified genes whose regulatory interactions were significantly altered in response to the knockout of *KAT2B* or *LAGE3*.

## Results

### Identification of cotinine target proteins and selection of research subjects

**Fig. 1:**
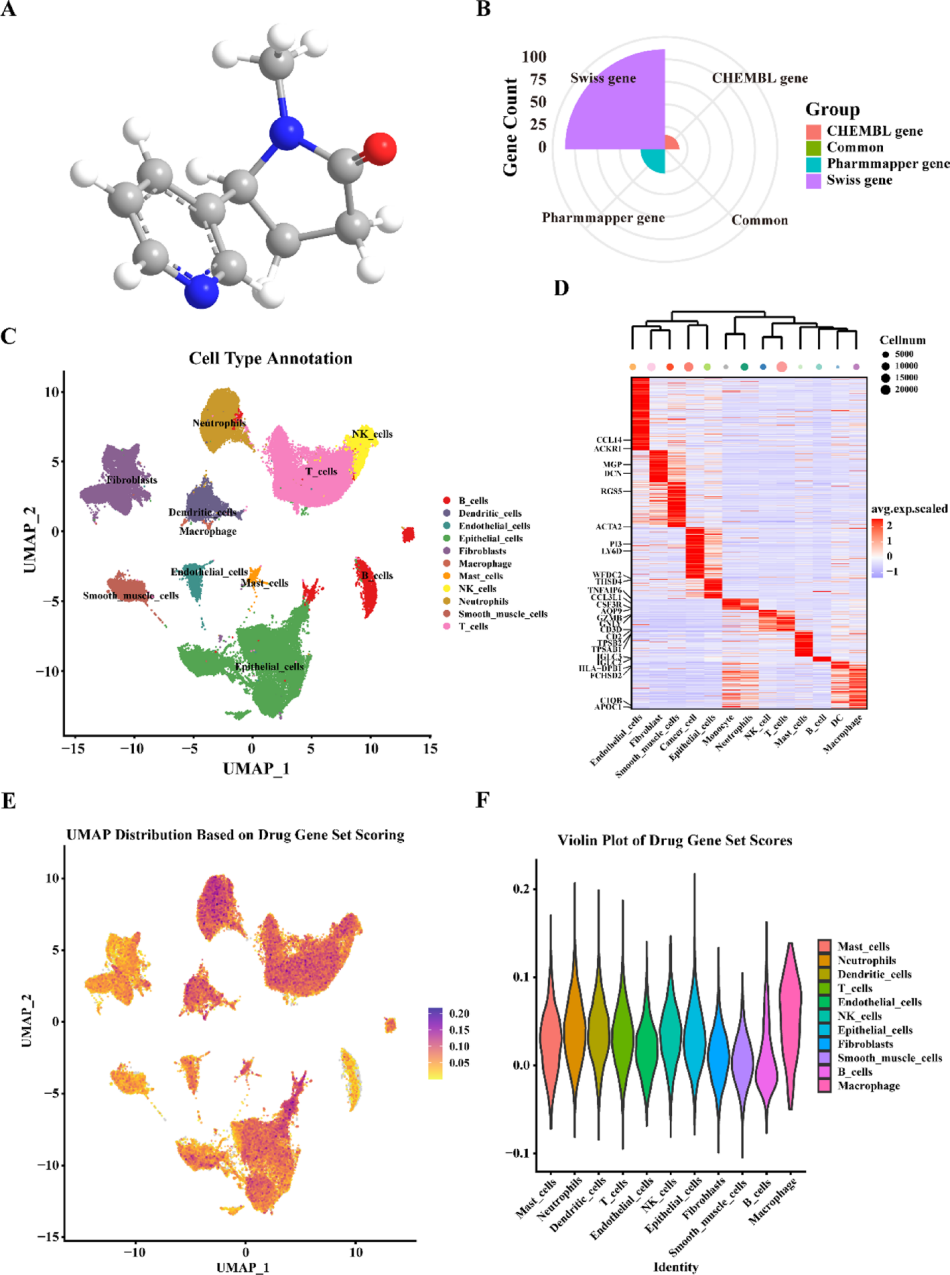
Identification of cotinine target proteins and selection of research subjects. **A** Three-dimensional structure of cotinine; **B** Petal diagrams displaying the number of genes predicted by each database; **C** UMAP two-dimensional distribution of individual cells; **D** Visualization of gene expression distribution across cells; **E** Visualization of cotinine-related gene expression for individual cells based on UMAP two-dimensional distribution; **F** Violin plot depicting cotinine-related gene expression across cell types

The molecular structure of cotinine was retrieved from the PubChem database, and its three-dimensional structure was obtained using Chem3D (Fig. 1A). Potential biological targets of cotinine were systematically predicted using three complementary databases: ChEMBL (curated bioactive molecules), PharmMapper (reverse pharmacophore mapping), and SwissTargetPrediction (ligand-based target prediction). Following data integration and deduplication, a total of 151 unique potential targets were identified (Fig. 1B). To investigate cellular heterogeneity, we performed single-cell RNA sequencing (scRNA-seq) followed by comprehensive bioinformatic analyses. The transcriptomic profiles of individual cells were visualized using Uniform Manifold Approximation and Projection (UMAP), a nonlinear dimensionality reduction technique. This analysis revealed distinct cellular subpopulations in the two-dimensional UMAP space, highlighting the intrinsic heterogeneity across cell clusters (Fig. 1C). To further characterize these subpopulations, we identified cell-type-specific marker genes and generated expression heatmaps for these markers. These marker genes not only validated the segregation of cell clusters but also provided insights into their unique functional identities (Fig. 1D). Based on the UMAP clustering and cell typing results, we integrated the 151 identified potential targets with single-cell expression data to calculate the expression set score of relevant genes in each cell. The resulting scores were compared across different cell types and mapped onto the UMAP plot, revealing significant differences in cotinine-related gene expression among distinct cell subpopulations (Fig. 1E). Violin plots of gene set scores for each cell type showed that epithelial cells exhibited the highest gene set scores, indicating that cotinine-related target gene expression activity is enriched in this cellular compartment (Fig. 1F). This observation suggests a testable hypothesis that epithelial cells may represent a primary cellular compartment through which cotinine exerts its biological effects in the cervical microenvironment. However, we emphasize that this computational finding alone does not establish epithelial cells as validated therapeutic targets; rather, it motivates the subsequent cell-type-specific experimental investigation that constitutes the core of this study. Importantly, cervical epithelial cells are known to be the primary cell type directly exposed to tobacco metabolites, as cotinine accumulates in cervical mucus at concentrations several-fold higher than in serum, and these cells serve as the cell-of-origin for the majority of cervical carcinomas. These biological considerations, together with the AUCell-based computational findings, provide a coherent rationale for the epithelial-cell-focused experimental design adopted in this work.

### Identification of cervical cancer-associated gene modules using hdWGCNA

**Fig. 2:**
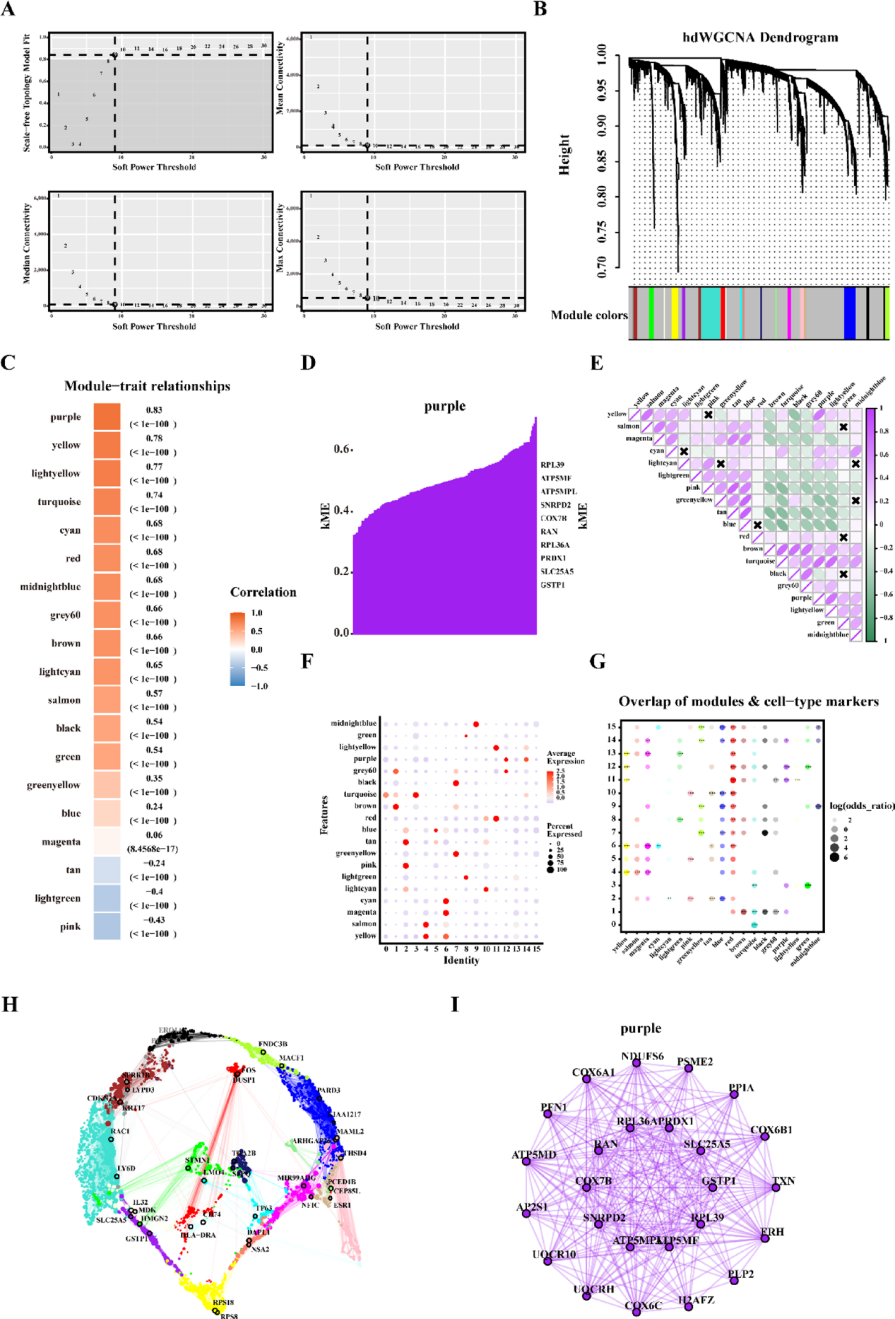
Identification of cervical cancer-associated gene modules using hdWGCNA. **A** Selection of soft threshold power to achieve scale-free topology; **B** Hierarchical clustering dendrogram displaying 19 distinct gene modules; **C** Module-trait relationship heatmap showing correlations between hdWGCNA-identified modules and sample traits (control vs. treated groups). Numbers inside and outside parentheses represent correlation coefficients and p-values, respectively; **D** Module significance analysis demonstrating gene expression and connectivity patterns. The vertical axis represents the eigengene expression profiles of each module, while the horizontal axis displays the topological overlap strength between genes; **E** Correlation analysis highlighting inter-module relationships; **F** Module-trait association analysis emphasizing enrichment patterns of modules; **G** Expression profiles of module genes across subgroups; **H** Visualization of module gene composition and their interrelationships; **I** Distribution patterns of hub genes in target modules

We employed the hdWGCNA software to identify key gene modules within cervical cancer-associated epithelial cells. As shown in Fig. 2A, we first constructed a gene co-expression network that conformed to a scale-free topology by selecting an optimal soft threshold power. Hierarchical clustering analysis identified 19 distinct gene modules with unique characteristics (Fig. 2B). Subsequent hdWGCNA module-trait relationship analysis revealed significant correlations between gene co-expression modules and sample phenotypes (control vs. treatment groups). The heatmap-annotated correlation coefficients (outside parentheses) and p-values (inside parentheses) demonstrated statistically significant associations between multiple modules and the treatment phenotype (Fig. 2C). Module significance analysis elucidated the expression patterns and interaction networks of intramodular genes (Fig. 2D). Further investigations revealed significant functional connections among these modules (Fig. 2E), with specific modules exhibiting marked enrichment in pathways related to abnormal proliferation (Fig. 2F). Subgroup expression profiling (Fig. 2G) and modular gene network visualization (Fig. 2H) uncovered expression heterogeneity of module genes across different clinical subgroups. The core gene composition and interaction networks within target modules were systematically characterized (Fig. 2I).

### Integrated multi-level analysis for target gene identification and pathway enrichment

**Fig. 3:**
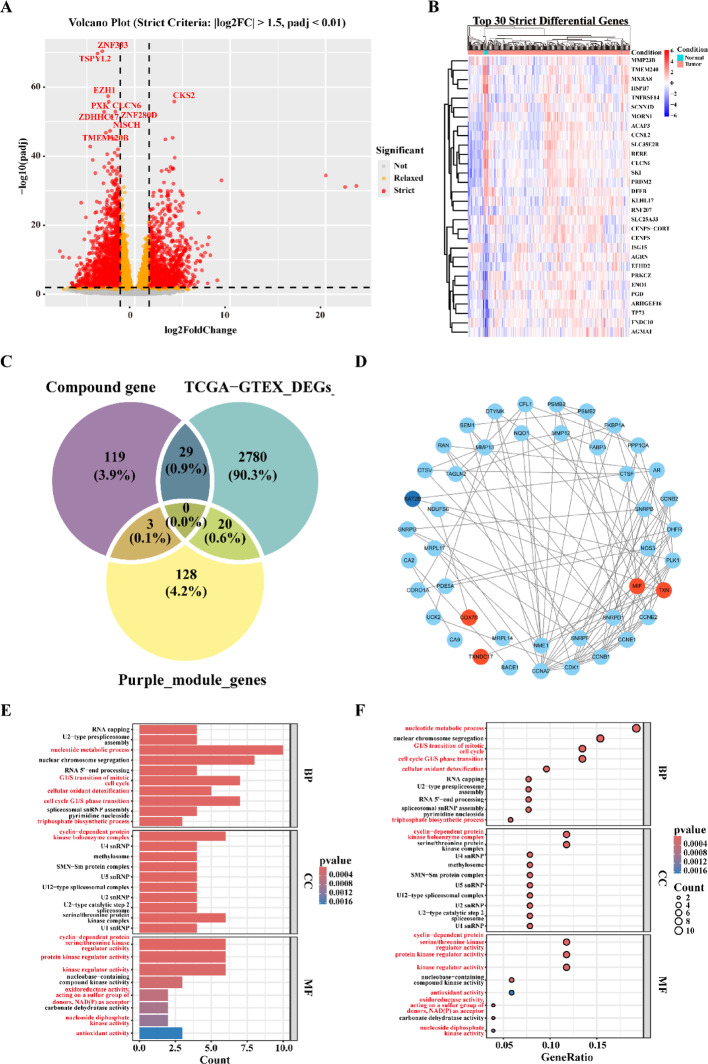
Multi-level analysis for identifying target genes. **A** Volcano plot displaying differentially expressed genes (DEGs) between control and cervical cancer pathological tissues based on log2 fold-change (logFC) and statistical significance; **B** Heatmap of DEG distribution showing the top 30 most significantly differentially expressed genes, with red and blue regions representing up- and down-regulated genes, respectively; **C** Venn diagram illustrating database-predicted target genes (purple), DEGs (light blue), and hdWGCNA module genes (yellow), with overlapping regions indicating genes identified by at least two methods; **D** Protein-protein interaction (PPI) network constructed from the 52 overlapping genes, whose visualization optimized with Cytoscape; **E-F** Gene Ontology (GO) enrichment analysis of potential target genes across biological processes (BP), cellular components (CC), and molecular functions (MF). **E** Bar plot with the x-axis indicating gene count and color gradient representing adjusted p-values (darker red indicates higher significance). **F** Dot plot with the x-axis showing gene ratio, color gradient denoting adjusted p-values (darker red reflects higher significance), and dot size corresponding to gene count

Differences in gene expression between the cervical cancer pathological group and the control group were analyzed using a volcano plot, with the screening criteria set as |log2FC| > 1.5 and *p* < 0.01. The horizontal axis represents log2 fold change (log2FC), while the vertical axis denotes -log10(p-value) (Fig. 3A). Subsequently, a heatmap of key gene expression profiles was generated to visualize the clustering of the 30 most significantly differentially expressed genes (normalized by row Z-score). Red regions indicate upregulated genes with high expression levels, whereas blue regions represent downregulated genes with low expression levels (Fig. 3B). A multi-method gene set intersection analysis was performed, and a Venn diagram was used to intuitively display the overlap among differentially expressed genes (DEGs, 2,829 genes, light blue), database-predicted target genes (151 genes, purple), and hdWGCNA module genes (151 genes, yellow). This analysis highlighted 52 genes co-selected by at least two methods (Fig. 3C). A protein–protein interaction (PPI) network was constructed for these 52 shared genes, where node size reflects the degree of connectivity. Key hub genes are speculated to be involved in the regulatory mechanisms underlying the development and progression of cervical cancer. The PPI network was further optimized for visualization using Cytoscape software (Fig. 3D). Systematic functional annotation and pathway enrichment analysis were conducted on the screened potential target genes. For Gene Ontology (GO) analysis, the overlapping genes were annotated from three dimensions: Biological Process (BP), Cellular Component (CC), and Molecular Function (MF) (Fig. 3E–F).

### Identification of core genes in the cotinine-induced cervical carcinogenesis process

**Fig. 4:**
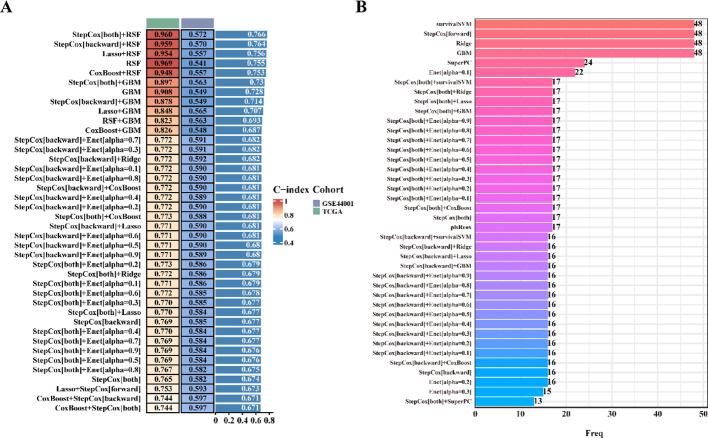
Identification of core genes in the cotinine-induced cervical carcinogenesis process. **A** Model performance comparison: Heatmap displaying the area under the curve (AUC) values of different models across multiple cohorts. The left column indicates the models, while the right column shows the corresponding AUC values (higher values indicate better performance). Color gradients represent different cohort sources; **B** Usage frequency comparison: Bar plot illustrating the utilization frequency of different models. Longer bars indicate higher usage frequency

Through comprehensive machine learning analysis of 52 candidate targets, we developed 150 predictive models to identify core genes involved in cotinine-associated cervical carcinogenesis. The StepCox[both] + GBM ensemble model demonstrated robust performance and accuracy during both training and validation phases (Fig. 4A-B). This approach identified 17 pivotal genes: *LAGE3*,* MIF*,* NQO1*,* CA9*,* FABP3*,* SNRPF*,* CORO1A*,* TCF7*,* SNRPB*,* KAT2B*,* DHFR*,* UCK2*,* CCNA2*,* FKBP1A*,* CCNB1*,* PSME2*, and *SNRPD1*.

### Transcriptome sequencing analysis of the effect of cotinine on H8 cells

**Fig. 5:**
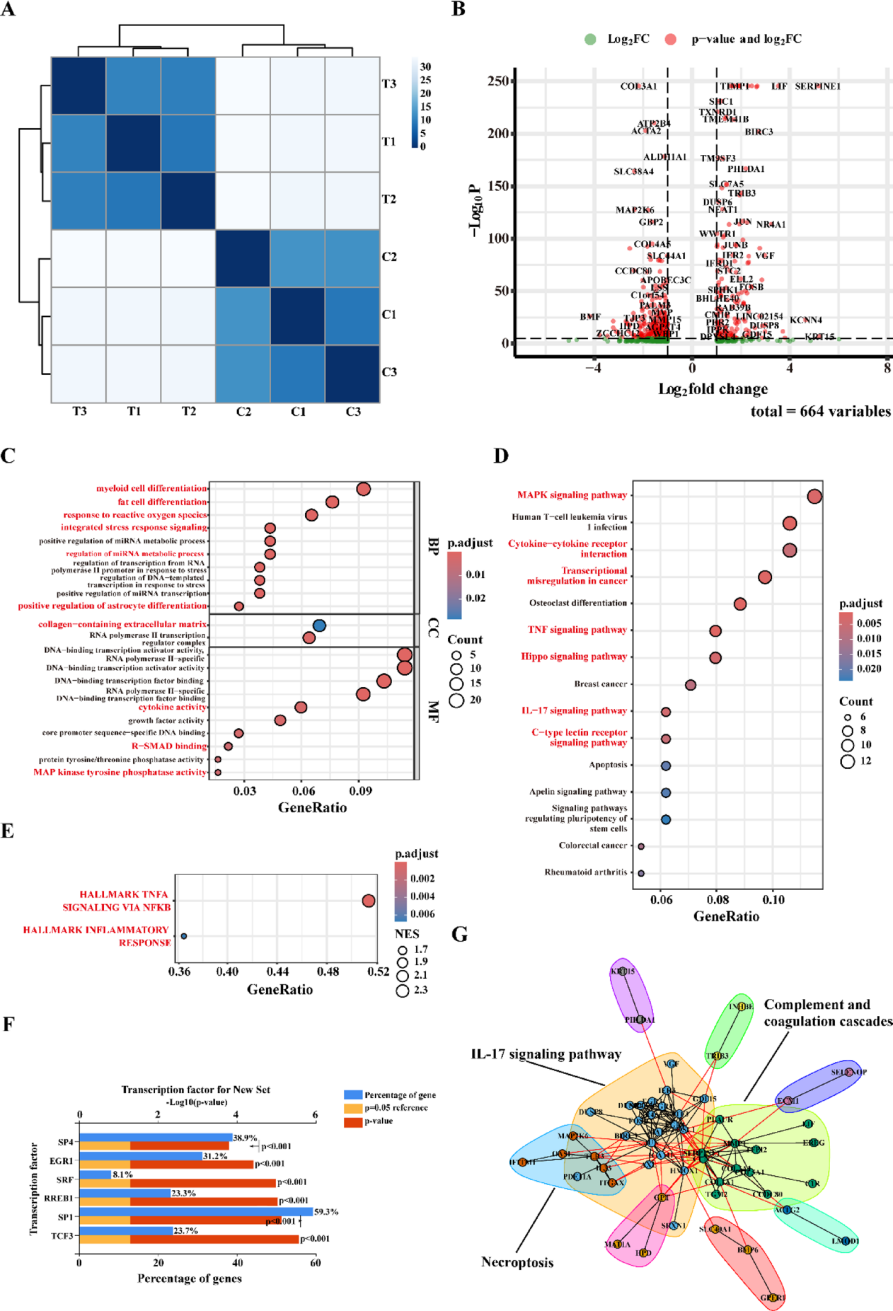
Transcriptome sequencing analysis of the effect of cotinine on H8 cells.** A** Heatmap of transcript expression correlation matrix for six samples (three control groups and three cotinine-treated groups); **B** Volcano plot displaying differentially expressed genes; **C-E** Bubble plots of GO, KEGG, and GSEA enrichment analyses for upregulated genes; **F** Transcription factor enrichment; **G** Protein-protein interaction network diagram

Transcriptome sequencing of cotinine-treated H8 cells identified 664 differentially expressed genes (231 upregulated, 433 downregulated). Principal component analysis (PCA) showed clear separation between the experimental and control groups (Fig. 5A–B). Functional annotation revealed a multi-pathway oncogenic network that includes several interconnected modules. First, it encompassed chronic inflammation driven by sustained activation of TNF and IL-17 signaling, accompanied by oxidative stress (ROS response) and impaired DNA repair via the integrated stress response. Second, the network involved differentiation abnormalities, including skewed myeloid cell differentiation, aberrant adipogenic programming, and dysregulated astrocyte differentiation mediated by R-SMAD–dependent TGF-β signaling. Third, it featured extracellular matrix (ECM) remodeling characterized by excessive collagen deposition and YAP-dependent Hippo pathway dysregulation, which promoted focal adhesion–mediated cell migration. Finally, the network comprised widespread signaling dysregulation, such as persistent MAPK activation, aberrant oncogene transcription, and disturbed miRNA metabolism (Fig. 5C–D). Gene set enrichment analysis (GSEA) and transcription factor enrichment analysis further validated the enrichment of inflammatory pathways (Fig. 5E–F). Protein–protein interaction (PPI) networks identified IL-17 signaling and necroptosis as central hub nodes (Fig. 5G). Together, these findings delineate the carcinogenic mechanism of cotinine in H8 cells and offer potential targets for therapeutic intervention.

### Transcriptome sequencing analysis of the effect of cotinine in HeLa cells

**Fig. 6:**
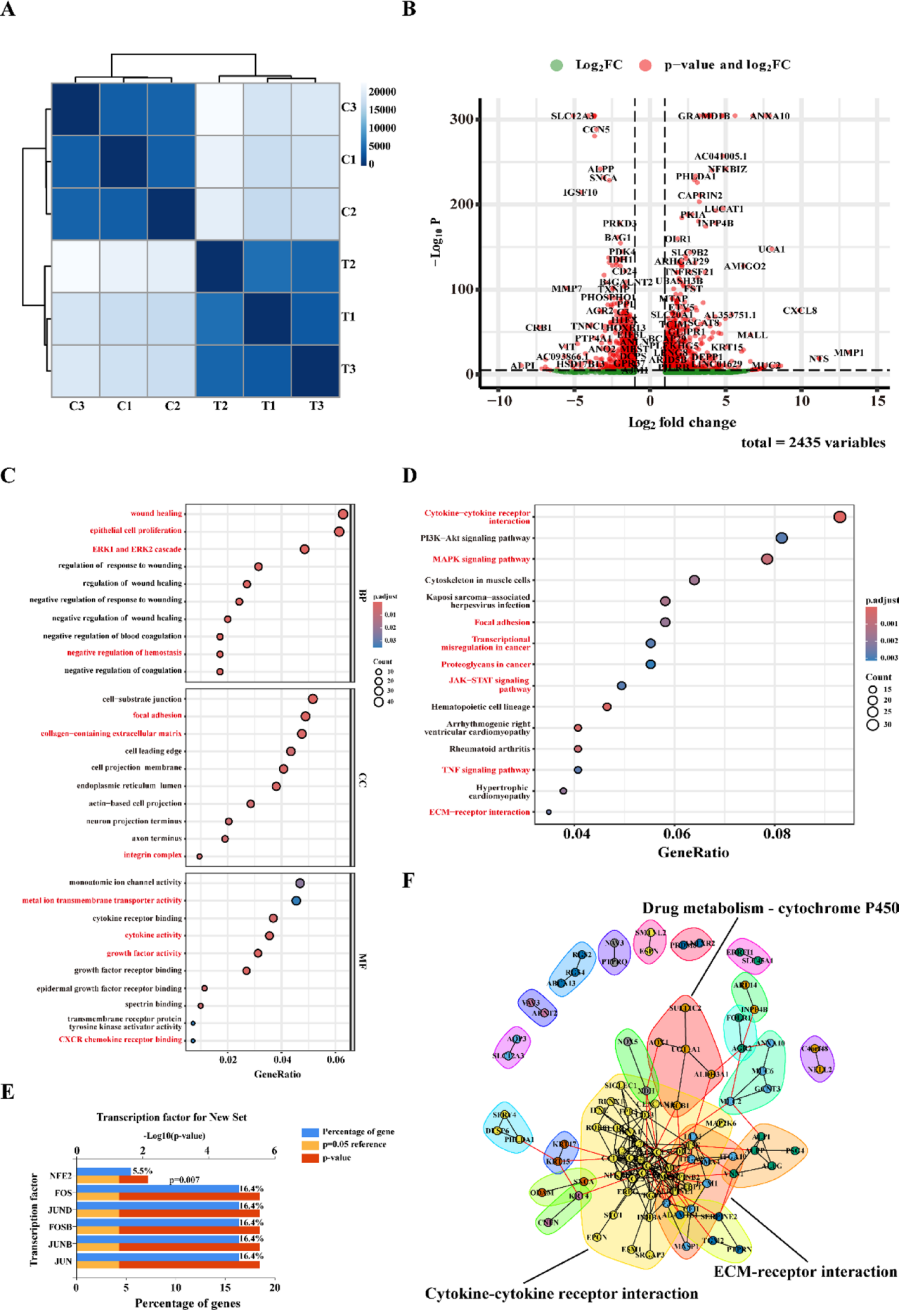
Transcriptome sequencing analysis of the effect of cotinine on HeLa cells. **A** Heatmap of transcript expression correlation matrix for six samples (three control groups and three cotinine-treated groups); **B** Volcano plot displaying differentially expressed genes; **C-D** Bubble plots of GO and KEGG enrichment analyses for upregulated genes; **E** Transcription factor enrichment; **F** Protein-protein interaction network diagram

Transcriptome profiling of cotinine‑exposed HeLa cells identified 2435 differentially expressed genes, of which 1424 were upregulated and 1011 were downregulated. Principal component analysis (PCA) revealed marked separation between the cotinine‑treated and control groups (Fig. 6A–B). Functional annotation uncovered a multi-stage toxicological cascade that links chronic cellular injury to cervical cancer progression and metastasis. This cascade included several key components: dysregulated proliferation driven by sustained ERK1/2 cascade activation and reinforced focal adhesion signaling, which subverts normal epithelial homeostasis into uncontrolled cell cycle progression through constitutive activation of wound-healing programs and defective repair termination, with ERK signaling further promoting telomerase-dependent immortalization. It also involved extracellular matrix (ECM) disorganization characterized by excessive collagen deposition, altered integrin complex conformation, MMP2/9-mediated basement membrane degradation, and impaired metal ion transmembrane transport leading to zinc/copper metabolic imbalance. Additionally, the cascade encompassed a pro-tumorigenic inflammatory microenvironment arising from disrupted cytokine–cytokine receptor crosstalk, TNF–JAK–STAT feedforward activation, CXCR-mediated recruitment of immunosuppressive cells, and cytokine-driven epithelial–mesenchymal transition (EMT) (Fig. 6C–D). Transcription factor enrichment results indicate a high correlation with cervical carcinogenesis (Fig. 6E). Protein–protein interaction (PPI) network analysis further highlighted key hub genes within the ECM–receptor interaction and cytokine–cytokine receptor interaction pathways (Fig. 6F). Collectively, these data delineate the mechanisms by which cotinine promotes the malignant progression and metastatic potential of HeLa cells, providing a mechanistic framework for the development of targeted therapeutic strategies.

### Molecular docking and molecular dynamics simulation on H8 cells

**Fig. 7:**
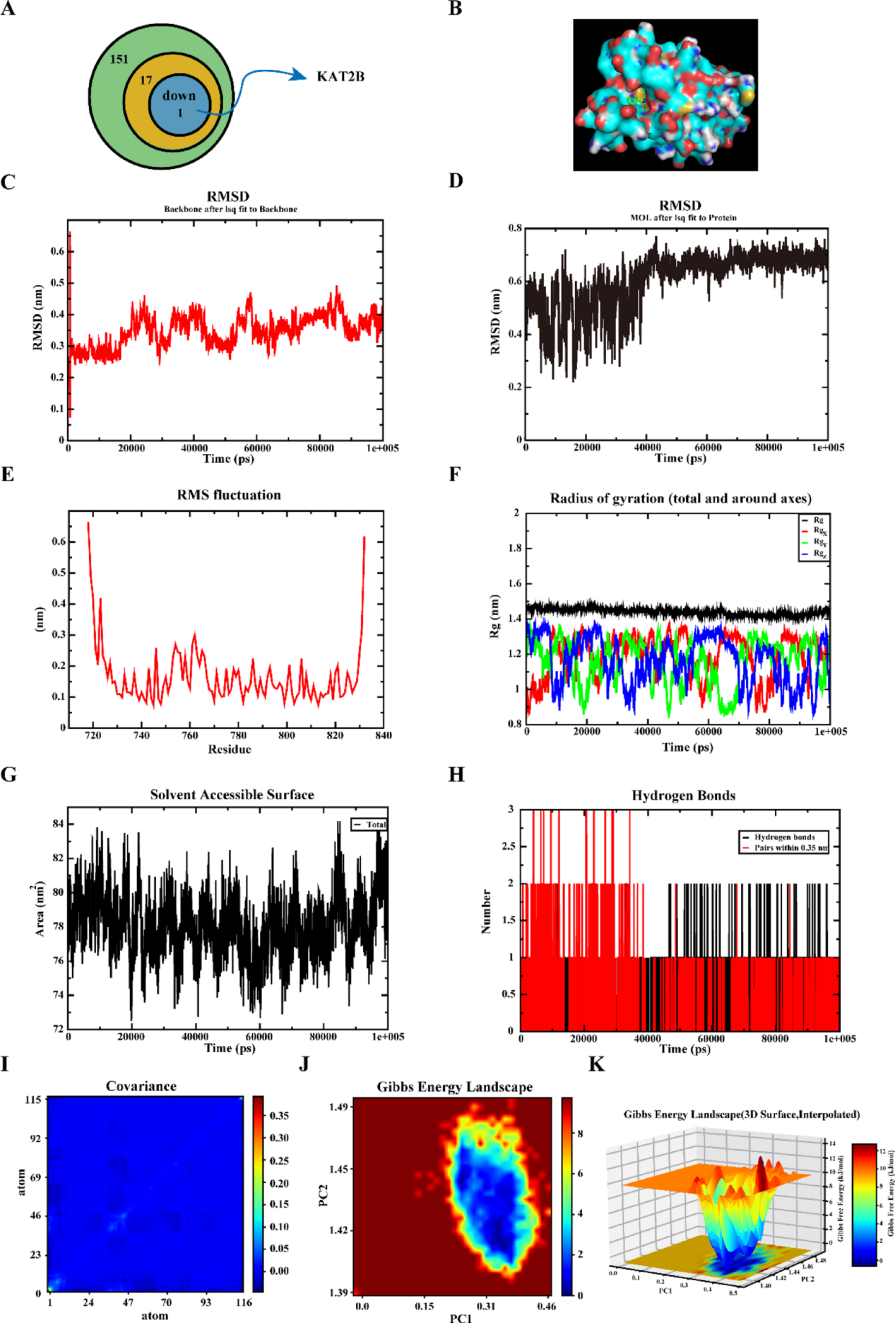
Molecular docking and molecular dynamics analysis of key targets on H8 cells. **A** Downregulated genes were identified from the 17 key targets obtained by machine learning based on transcriptomic analysis; **B** Molecular docking results; **C-D** Root-mean-square deviation (RMSD) curves of protein-ligand complexes; **E** Root-mean-square fluctuation (RMSF) analysis at the protein residue level; **F** Radius of gyration curves of protein-ligand complexes; **G** Protein conformational adjustment and its interaction with solvent; **H** Dynamic changes in the number of hydrogen bonds between protein and ligand; **I** Covariance matrix heatmaps of protein residue movements; **J** Two-dimensional Gibbs free energy landscape based on principal component analysis; **K** Three-dimensional Gibbs free energy landscape based on principal component analysis

Based on transcriptome analysis results, we further validated the 17 key targets identified by machine learning, ultimately confirming KAT2B as the core target mediating cotinine-induced effects in H8 cells (Fig. 7A). To verify the reliability of KAT2B as the selected key target, molecular docking analysis was performed. All docked conformations exhibited high binding affinities, with the optimal conformation showing a binding energy of −5.5 kcal/mol, indicating that cotinine can form stable complexes with KAT2B (Fig. 7B).

Subsequently, molecular dynamics (MD) simulations were carried out to assess the dynamic stability of the cotinine-KAT2B complex. Root-mean-square deviation (RMSD) analysis revealed that after 40,000 picoseconds (ps) of simulation, the backbone conformation of KAT2B was fully relaxed and maintained stability, while the ligand binding mode reached dynamic equilibrium (Fig. 7C-D). Root-mean-square fluctuation (RMSF) results demonstrated high flexibility in the N/C termini (residues 720 and 820–840) and surface loop regions (near residues 760 and 800), whereas the core structural domain (residues 740–780) displayed high rigidity. This observation is consistent with the general structural principle of proteins, namely “flexible termini and rigid core” (Fig. 7E).

Moreover, the total radius of gyration (Rg) of KAT2B was stably maintained at 1.4–1.5 nm with minimal fluctuations; Rg values along each axis only oscillated within the equilibrium range, suggesting that the protein retained an overall compact conformation without obvious unfolding or expansion. Notably, Rg fluctuations along the Z-axis were significantly greater than those along the X/Y axes, indicating that KAT2B adopted a non-spherical structure and that the Z-axis was the primary direction of structural flexibility (Fig. 7F).

The solvent-accessible surface area (SASA) remained stable at 76–84 nm², oscillating frequently within the equilibrium range throughout the simulation without sustained upward or downward trends. This finding demonstrated that the surface exposure of KAT2B was stable, with no significant structural extension or folding (Fig. 7G). At the ligand binding force level, one core effective hydrogen bond was stably maintained during the entire simulation period, while potential hydrogen bond pairs underwent frequent breakage and reformation—consistent with the dynamic nature of hydrogen bonds—which provides critical support for the stability of the cotinine-KAT2B complex (Fig. 7H).

The covariance heatmap showed no abnormally negatively correlated or extremely positively correlated regions, indicating that atomic motion patterns during the simulation were natural and stable, and the acquired data were reliable (Fig. 7I). Additionally, two-dimensional (2D) and three-dimensional (3D) Gibbs free energy landscape (FEL) plots collectively delineated the dynamic binding pathway of the small molecule cotinine to KAT2B (Fig. 7J-K).

### Molecular docking and molecular dynamics simulation in HeLa cells

**Fig. 8:**
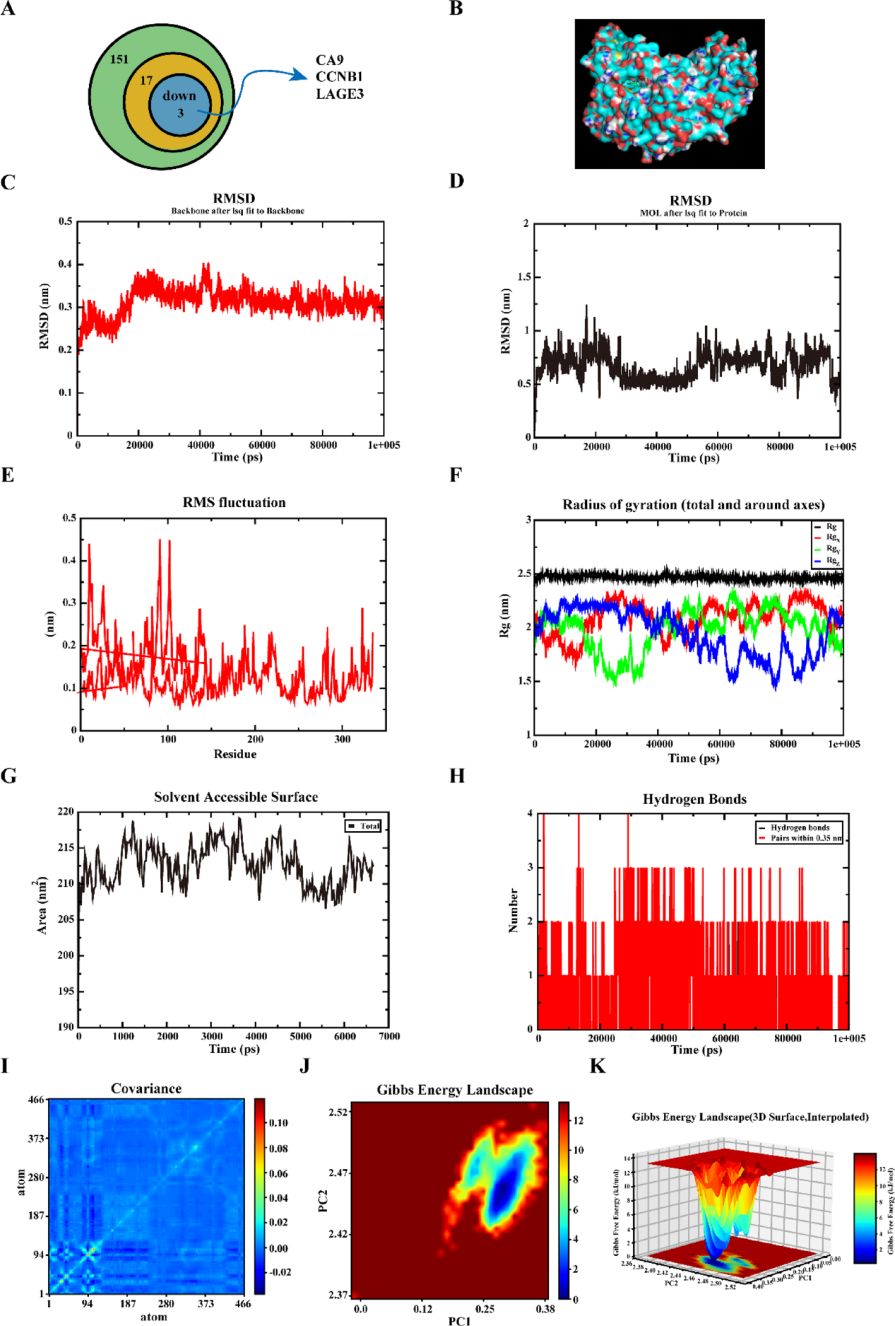
Molecular docking and molecular dynamics analysis of key targets on HeLa cells. **A** Downregulated genes were identified from the 17 key targets obtained by machine learning based on transcriptomic analysis; **B** Molecular docking results; **C-D** Root-mean-square deviation (RMSD) curves of protein-ligand complexes; **E** Root-mean-square fluctuation (RMSF) analysis at the protein residue level; **F** Radius of gyration curves of protein-ligand complexes; **G** Protein conformational adjustment and its interaction with solvent; **H** Dynamic changes in the number of hydrogen bonds between protein and ligand; **I** Covariance matrix heatmaps of protein residue movements; **J** Two-dimensional Gibbs free energy landscape based on principal component analysis; **K** Three-dimensional Gibbs free energy landscape based on principal component analysis

Drawing on transcriptome sequencing data, we further validated the 17 key targets screened via machine learning, and ultimately pinpointed CA9, CCNB1, and LAGE3 as core targets that mediate cotinine’s effects on HeLa cells (Fig. 8A). Guided by relevant literature, we hypothesized that LAGE3 is closely linked to cervical cancer progression. To confirm the suitability of LAGE3 as the key target for subsequent analyses, molecular docking experiments were conducted. All docked conformations showed robust binding affinities, and the optimal conformation exhibited a binding energy of −6.2 kcal/mol, verifying that cotinine can form stable complexes with LAGE3 (Fig. 8B). To further characterize the dynamic stability of the cotinine-LAGE3 complex, molecular dynamics (MD) simulations were performed. Root-mean-square deviation (RMSD) analysis showed that after 20,000 picoseconds (ps) of simulation, the protein backbone conformation achieved full relaxation and maintained stability, while the ligand binding mode reached dynamic equilibrium (Fig. 8C-D). Root-mean-square fluctuation (RMSF) analysis indicated high flexibility in the N/C termini and surface loop regions (e.g., near residue 100), whereas the core structural domain exhibited high rigidity—aligning with the well-recognized “flexible termini and rigid core” characteristic of protein structures (Fig. 8E).

Furthermore, the total radius of gyration (Rg) of LAGE3 was stably sustained at 2.45–2.50 nm with negligible fluctuations; Rg values along each axis only varied within the equilibrium range, implying that the protein retained an overall compact structure without significant unfolding or expansion. Of note, Rg fluctuations along the Z-axis were markedly higher than those along the X/Y axes, suggesting that LAGE3 adopted a non-spherical conformation and that the Z-axis was the primary direction of structural flexibility (Fig. 8F). The solvent-accessible surface area (SASA) reached dynamic equilibrium (207–219 nm²) after 1,000 ps of simulation, with stable surface exposure and no extreme structural extension or folding (Fig. 8G). Regarding ligand binding forces, one core effective hydrogen bond was stably preserved throughout the simulation period, while potential hydrogen bond pairs underwent frequent dissociation and reformation—consistent with the intrinsic dynamic properties of hydrogen bonds—which contributes crucial support to the stability of cotinine-LAGE3 binding (Fig. 8H). The covariance heatmap exhibited no abnormally negative or extremely positive correlation regions, indicating that atomic motion patterns during the simulation were natural and stable, and thus the resulting data were credible (Fig. 8I). Additionally, two-dimensional (2D) and three-dimensional (3D) Gibbs free energy landscape (FEL) diagrams jointly illustrated the dynamic binding process of the small molecule cotinine to LAGE3 (Fig. 8J-K).

### In silico knockout analysis of single-cell data from normal epithelial cells

**Fig. 9:**
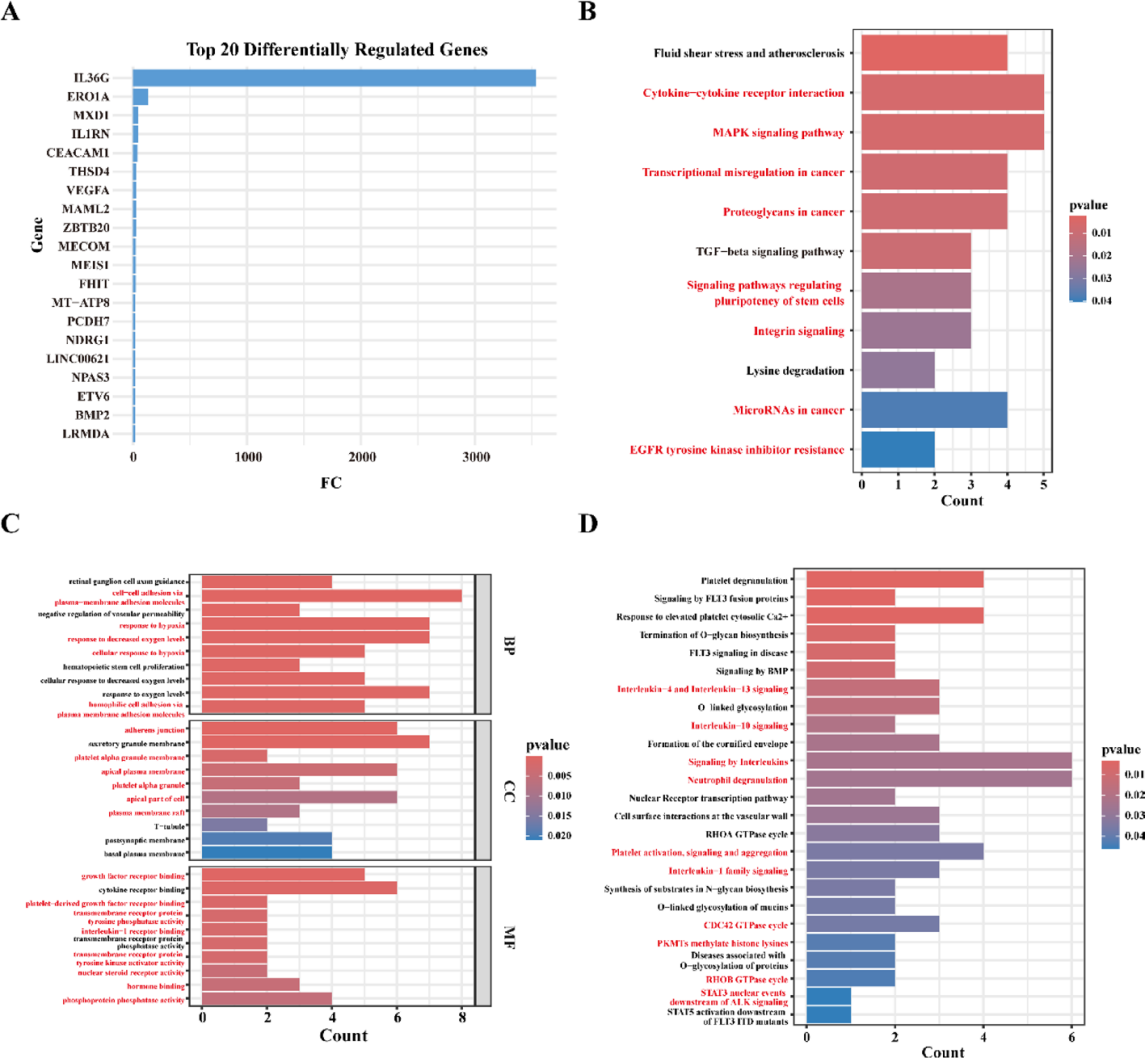
In silico knockout analysis of single-cell data from normal epithelial cells. **A** Barplot of the top 20 differentially regulated genes; **B-D** Barplots of GO, KEGG and Reactome enrichment analyses for stable knockout-responsive genes

To investigate the downstream effects of *KAT2B* deletion, we performed in silico knockout using scTenifoldKnk and identified the top 20 stable knockout-responsive genes with the most significant expression alterations (Fig. 9A). Integrating Gene Ontology (GO), Kyoto Encyclopedia of Genes and Genomes (KEGG), and Reactome pathway analyses, we delineated a multi-pathway oncogenic network consisting of four core functional modules: First, chronic inflammatory activation driven by upregulated cytokine-cytokine receptor interactions and interleukin-related signaling cascades, accompanied by oxidative stress and defective DNA repair, which collectively establish a pro-tumorigenic microenvironment. Second, aberrant cellular differentiation mediated by dysregulated R-SMAD-dependent TGF-β signaling and abnormal STAT3 nuclear translocation, which promoted proliferative disorders and immune evasion. Third, extracellular matrix (ECM) remodeling and migratory activation involving disrupted integrin signaling, dysregulated RHOA/CDC42 GTPase cycling, and impaired cell-cell adhesion, which synergistically enhance the invasive and migratory capacities of cells. Fourth, global signaling dysregulation characterized by sustained MAPK activation, cancer-associated transcriptional and microRNA (miRNA) dysregulation, aberrant growth factor receptor binding, and impaired tyrosine kinase activity, which drives uncontrolled cell proliferation and oncogenic transformation (Fig. 9B–D).

### In silico knockout analysis of single-cell data from malignant epithelial cells

**Fig. 10:**
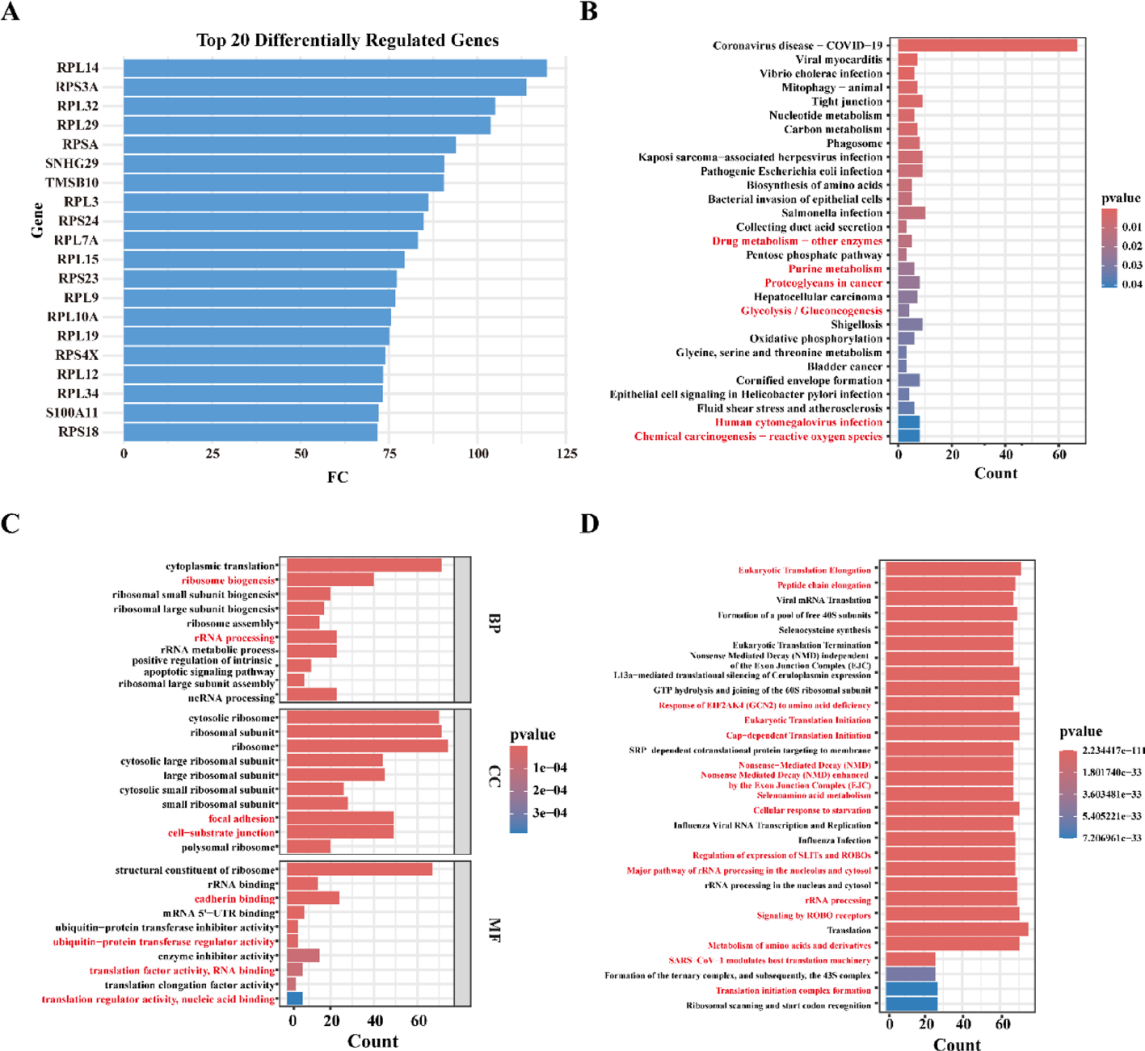
In silico knockout analysis of single-cell data from malignant epithelial cells. **A** Barplot of the top 20 differentially regulated genes; **B-D** Barplots of GO, KEGG and Reactome enrichment analyses for stable knockout-responsive genes

To explore the oncogenic mechanisms underlying *LAGE3* deletion, we utilized scTenifoldKnk for in silico gene knockout and prioritized the top 20 responsive genes with the most robust expression alterations (Fig. 10A). Functional enrichment analyses, including Gene Ontology (GO), Kyoto Encyclopedia of Genes and Genomes (KEGG), and Reactome pathways, uncovered a complex oncogenic network regulated by *LAGE3* deletion, which drives cervical cancer progression through interconnected molecular mechanisms. Specifically, these mechanisms involve three key facets: first, malignant transformation mediated by dysregulated translation, perturbed ribosome biogenesis, and impaired p53-dependent apoptotic signaling, thereby facilitating uncontrolled cell proliferation and immortalization; second, enhanced invasion and metastasis via aberrant focal adhesion formation, dysregulated integrin/focal adhesion kinase (FAK) signaling, and matrix metalloproteinase (MMP)-dependent extracellular matrix (ECM) remodeling, all of which promote epithelial-mesenchymal transition (EMT) and cell migration; third, metabolic reprogramming supported by hyperactivated glycolysis, disrupted nucleotide and amino acid metabolism, and augmented stress response pathways, enabling cancer cells to survive under nutrient deprivation and oxidative stress conditions (Fig. 10B–D).

## Discussion

Cervical cancer remains a significant global public health challenge, with smoking identified as a major risk factor for its carcinogenesis [26]. Cotinine, a widely used biomarker for verifying smoking status, has emerged as a key mediator linking tobacco exposure to cervical cancer progression, yet the precise molecular mechanisms underlying its carcinogenic effects remain incompletely elucidated [8, 27]. Leveraging integrated multi-omics technologies combined with molecular biology validation, this study systematically delineated the dual carcinogenic mechanisms of cotinine in cervical cancer: cotinine promotes tumor initiation by targeting *KAT2B*, a critical molecular hub in epithelial cells, and specifically modulates key malignant cell targets (*CA9*, *CCNB1*, and *LAGE3*) to accelerate cancer progression and metastatic dissemination.

### Research background and rationale

Numerous studies have confirmed a robust statistical association between tobacco smoke exposure and cervical cancer susceptibility, but the systemic pathogenic pathways connecting these two factors remain fragmented [28]. Existing evidence suggests that nicotine, the primary addictive component of tobacco, can promote tumor progression, and chronic smoking may induce carcinogenesis via immune function impairment. However, most prior research has focused on single linear pathway analyses, failing to construct a comprehensive “exposure-response-effect” regulatory network and overlooking the dynamic crosstalk between genomic damage and immune microenvironment remodeling [29, 30]. To address this critical research gap, we innovatively adopted a triangular systems toxicology strategy, establishing a multidimensional framework encompassing computational prediction, machine learning prioritization, and experimental validation to decipher cotinine-driven carcinogenic mechanisms in cervical cancer.

### KAT2B-mediated carcinogenic pathways

*KAT2B* serves as a pivotal target of cotinine, with its downregulation contributing to multiple oncogenic processes in cervical cancer. Previous studies have demonstrated that *KAT2B* depletion induces genomic instability, inhibits the efficient acetylation of seven lysine residues within the chromatin-binding motif of the tumor suppressor PALB2, and reduces PALB2-nucleosome binding capacity [31]. This impairment ultimately blocks RAD51-mediated homologous recombination-dependent DNA damage repair and fails to protect active genes from replication stress, creating a genomic landscape conducive to tumorigenesis. Consistently, our transcriptomic analysis in cotinine-treated H8 cells revealed impaired DNA repair signatures under the integrated stress response, directly linking KAT2B downregulation to defective homologous recombination repair in cervical carcinogenesis. Additionally, knockout of *KAT2A* and *KAT2B* impairs mitochondrial function, leading to mitochondrial double-stranded RNA (dsRNA) accumulation and triggering a robust interferon response [32]. In parallel, our data identified oxidative stress (ROS response) and chronic inflammation driven by sustained TNF and IL-17 signaling activation in cotinine-exposed H8 cells, corroborating that KAT2B loss propagates inflammatory cascades as a key initiator of tumor-promoting microenvironment. As a key initiator of inflammatory signaling, *KAT2B* downregulation also induces chronic inflammation, a well-established driver of tumor progression. Furthermore, genetic or pharmacological inactivation of *KAT2B* disrupts epithelial-mesenchymal transition (EMT) and diminishes cellular stemness, fostering a microenvironment that supports tumor cell migration and survival during malignant transformation [33]. This mechanism was further validated in our HeLa cell transcriptome, where cotinine exposure drove cytokine-driven EMT, MMP2/9-mediated basement membrane degradation, and ECM disorganization characterized by excessive collagen deposition and altered integrin complex conformation, providing direct evidence that KAT2B downregulation orchestrates a pro-metastatic transcriptional program. Collectively, our multi-omics profiling not only recapitulates each of these previously established KAT2B-dependent pathways but also demonstrates their concurrent activation by cotinine in both premalignant H8 and malignant HeLa cells, grounding our observations in validated mechanistic frameworks.

### LAGE3-driven malignant progression

*LAGE3*, a core component of the KEOPS complex, is another key target of cotinine, with its dysregulation contributing to cervical cancer progression through multiple mechanisms [34]. The KEOPS complex is essential for telomere maintenance, transcriptional regulation, and t6A modification (N⁶-threonylcarbamoyladenosine) of tRNAs, and all subunits—including *LAGE3*—are required for its functional integrity [35]. Downregulation of *LAGE3* disrupts KEOPS complex assembly, impairing the tRNA t6A modification—a critical process for maintaining normal tRNA function. Defects in t6A modification lead to protein translation errors, ribosome stalling, and protein aggregation, which sequentially induce DNA replication stress and double-strand breaks, further exacerbating genomic instability [36]. In direct alignment with this mechanism, our cotinine-treated H8 cells exhibited impaired DNA repair under integrated stress response, while HeLa cells showed sustained ERK1/2 cascade activation and defective repair termination that subverts normal epithelial homeostasis into uncontrolled cell cycle progression. Moreover, *LAGE3* and *OSGEP* (another KEOPS subunit) are specifically enriched at the promoters and enhancers of active genes; thus, *LAGE3* downregulation disrupts transcriptional programs, increases cellular susceptibility to transcriptional stress, and promotes the production of aberrant transcripts, facilitating tumor heterogeneity and malignant progression [35]. In our data, cotinine exposure induced aberrant oncogene transcription and disturbed miRNA metabolism in H8 cells, as well as dysregulated proliferation driven by sustained ERK1/2 cascade activation and reinforced focal adhesion signaling in HeLa cells, phenocopying the transcriptional stress phenotype and extending it to cervical cancer. Notably, *LAGE3* downregulation also enhances CD8⁺ T cell infiltration and upregulates immune checkpoint molecule expression, establishing a malignant microenvironment characterized by genomic instability, persistent cellular stress, and extracellular matrix (ECM) abnormalities—all of which promote cytokine storms and immunosuppressive cell recruitment to sustain tumor growth [37]. This finding is strongly supported by our transcriptomic data, which revealed a pro-tumorigenic inflammatory microenvironment arising from disrupted cytokine–cytokine receptor crosstalk, TNF–JAK–STAT feedforward activation, and CXCR-mediated recruitment of immunosuppressive cells in cotinine-exposed HeLa cells, as well as chronic TNF/IL-17-driven inflammation in H8 cells. Together, our integrative analysis demonstrated that cotinine-driven LAGE3 downregulation recapitulates and connects each of these previously reported KEOPS-related pathogenic mechanisms—translational infidelity, transcriptional stress, genomic instability, and immune remodeling—within a unified cervical carcinogenesis model.

### Comparative analysis of cotinine toxicity in H8 and HeLa cells

A critical oversight in prior research is the failure to recognize that environmental toxins can exert stage-specific toxic effects on the same tissue or cell type. Most existing studies have focused on structural and functional changes in a single cellular stage following toxin exposure, rather than comparing effects across different cellular states relevant to carcinogenesis. For example, studies on smoking-induced effects in H8 cells have shown that nicotine promotes H8 cell proliferation in vitro via activation of the RPS27a-Mdm2-p53 pathway and the AKT/mTOR-4EBP1/eIF4E-oncoprotein (c-Myc, CyclinD1, etc.) axis [38, 39]. In contrast, research on HeLa cells has focused on nicotine-induced enhancement of cell migration and invasion via the PI3K/Akt/NF-κB pathway [40]. Importantly, these existing studies have focused exclusively on nicotine rather than its major metabolite cotinine, which has a longer half-life and distinct pharmacokinetic profile in chronic smokers [8]. To date, no studies have explored whether smoking or its metabolites can simultaneously affect both H8 (cervical epithelial) and HeLa (cervical cancer) cells. This study addresses this gap by conducting a comparative analysis of cotinine (the major metabolite of nicotine) toxicity in H8 and HeLa cells under identical culture conditions. Our transcriptomic profiling (Sect. 3.5 and 3.6) provides the first direct quantitative comparison of cotinine-induced responses across both cell types. Our findings reveal that cotinine induces a multi-pathway synergistic carcinogenic response in H8 cells, involving chronic inflammation (TNF and IL-17 signaling, ROS response), differentiation abnormalities (TGF-β/R-SMAD-mediated), ECM remodeling (YAP-dependent Hippo dysregulation, excessive collagen deposition), and signaling pathway dysregulation (persistent MAPK activation, disturbed miRNA metabolism). Notably, none of these H8-specific mechanisms—particularly the YAP-dependent Hippo dysregulation and TGF-β/R-SMAD-mediated differentiation abnormalities—have been previously reported in any smoking- or nicotine-related study. In HeLa cells, cotinine targets key molecular nodes to induce uncontrolled proliferation (sustained ERK1/2 cascade activation, defective repair termination), metal ion transport dysfunction (zinc/copper metabolic imbalance), and cytokine storms (TNF-JAK-STAT feedforward activation, CXCR-mediated immunosuppression), ultimately accelerating cancer progression and metastasis. The identification of metal ion transport dysfunction as a cotinine-elicited event in malignant cells has no precedent in the tobacco-associated cervical cancer literature. Furthermore, while prior HeLa-focused studies have described nicotine-induced PI3K/Akt/NF-κB activation [40], our data revealed that cotinine activates a distinct but partially overlapping repertoire—centered on ERK1/2 and JAK-STAT pathways—suggesting that nicotine and its metabolite may exert non-identical oncogenic effects. Collectively, this comparative analysis demonstrated that our study provides the first stage-specific framework reconciling previously fragmented observations from H8-only or HeLa-only studies into a unified toxicological model of cotinine-driven cervical carcinogenesis.

### Research innovation and limitations

The innovation of this study lies in its adoption of a systems toxicology approach to decipher cotinine-driven cervical carcinogenesis, overcoming the limitations of single-pathway analyses to construct a comprehensive regulatory network. By integrating network toxicology, single-cell analysis, multi-omics data, and experimental validation, we identified *KAT2B* and *LAGE3* as core targets of cotinine and elucidated their multi-faceted oncogenic mechanisms, providing new insights into the link between smoking and cervical cancer. Additionally, our comparative analysis of H8 and HeLa cells offers a stage-specific perspective on cotinine toxicity, highlighting distinct carcinogenic mechanisms in normal versus malignant cervical cells.

Despite these contributions, the study has several limitations. First, it lacks protein-level validation of cotinine-induced changes in *KAT2B* and *LAGE3* expression and function, relying primarily on transcriptomic data. Second, in vivo functional experiments are needed to confirm the causal role of *KAT2B* and *LAGE3*, as well as key downstream pathways, in cotinine-driven cervical carcinogenesis. Third, the study does not address clinical stratification of samples, which may limit the generalizability of our findings to diverse patient populations.

### Future prospects

While this study provides comprehensive computational and transcriptomic evidence for the cell-type-specific carcinogenic mechanisms of cotinine in cervical cancer, several important directions warrant future investigation.

First, experimental validation of the identified core targets (KAT2B, CA9, CCNB1, LAGE3) through CRISPR/Cas9-mediated knockout and siRNA-mediated knockdown in both H8 and HeLa cells, as well as in additional cervical cancer cell lines (e.g., SiHa, CaSki, C33A), would provide direct functional evidence for their roles in cotinine-induced carcinogenesis. Rescue experiments with target gene overexpression would further confirm the specificity of the observed phenotypic effects.

Second, in vivo validation using animal models is essential for translational relevance. Cotinine exposure experiments in xenograft mouse models or transgenic models with conditional knockout of the identified targets in cervical epithelium would provide critical pharmacokinetic and pharmacodynamic evidence that cannot be obtained from in vitro systems alone.

Third, the four validated targets may hold clinical translational potential as candidate biomarkers for risk stratification in tobacco-exposed female populations. Prospective cohort studies measuring the expression or activity of KAT2B, CA9, CCNB1, and LAGE3 in cervical cytology specimens from smokers versus non-smokers could evaluate their utility as early indicators of tobacco-associated cervical carcinogenesis. Furthermore, the cotinine-target binding modes characterized by our molecular dynamics simulations could inform structure-based drug design for the development of small-molecule antagonists that competitively inhibit cotinine binding to these targets.

Fourth, future integration of spatial transcriptomics and proteomics data would enable validation of the cell-type-specific mechanisms at the tissue architecture level. While our scRNA-seq-based analysis revealed cell-type-specific expression patterns, spatial context—including paracrine signaling between epithelial cells and the tumor microenvironment—is critical for understanding the full complexity of cotinine-induced cervical carcinogenesis in situ.

Fifth, extended investigation into other tobacco-specific nitrosamines (e.g., NNK, NNN) and their metabolites using similar systems toxicology frameworks would provide a more comprehensive understanding of tobacco-associated cervical carcinogenesis beyond the effects of cotinine alone. The combinatorial effects of multiple tobacco-derived compounds on the cervical microenvironment represent an important but underexplored area of research.

Finally, the computational-experimental triangular framework established in this study could be adapted for investigating the toxicological mechanisms of other environmental risk factors for cervical cancer (e.g., prolonged oral contraceptive use, high parity), thereby contributing to a systems-level understanding of how multiple environmental exposures converge on shared molecular pathways to promote cervical carcinogenesis.

## Conclusions

This study systematically reveals, through network toxicology, multi-omics technologies, molecular biology experiments, molecular docking technology and molecular dynamics simulation, that cotinine acts on key molecular targets *KAT2B* in epithelial cells promoting tumorigenesis, while simultaneously cotinine acts on key molecular targets *CA9*, *CCNB1* and *LAGE3* in malignant cells triggering toxicological effects and ultimately accelerating cancer progression and metastasis. This discovery not only elucidates the dual carcinogenic mechanism of cotinine but also provides novel molecular targets and intervention strategies for the prevention and treatment of cervical cancer.

## Supplementary Information

Supplementary Material 1.

## Data Availability

Data supporting the findings of this study are available from the corresponding author upon reasonable request.

## Abbreviations

AUC: Area under the curve
BP: Biological process
CA9: Carbonic anhydrase 9
CC: Cellular component
CCNB1: Cyclin B1
CESC: Cervical squamous cell carcinoma and endocervical adenocarcinoma
DDR: DNA damage response
DEG: Differentially expressed gene
ECM: Extracellular matrix
EMT: Epithelial-mesenchymal transition
Enet: Elastic net
FBS: Fetal bovine serum
GBM: Gradient boosting machine
GEO: Gene expression omnibus
GO: Gene ontology
GSEA: Gene set enrichment analysis
hdWGCNA: High-dimensional weighted gene co-expression network analysis
HPV: Human Papillomavirus
KAT2B: Lysine Acetyltransferase 2B
KEGG: Kyoto encyclopedia of genes and genomes
LAGE3: L antigen family member 3
LASSO: Least absolute shrinkage and selection operator
LDA: Linear discriminant analysis
log2FC: log2 fold change
MD: Molecular dynamics
MF: Molecular function
MMP: Matrix metalloproteinase
PCA: Principal component analysis
PPI: Protein-protein interaction
RF: Random forest
RMSD: Root-mean-square deviation
RMSF: Root-mean-square fluctuation
RNA-Seq: RNA sequencing
ROC: Receiver operating characteristic
ROS: Reactive oxygen species
SASA: Solvent-accessible surface area
scRNA-seq: Single-cell RNA sequencing
STRING: Search tool for the retrieval of interacting genes/proteins
SVM: Support vector machine
TCGA: The Cancer Genome Atlas
TCR: T cell receptor
TOM: Topological overlap matrix
UMAP: Uniform manifold approximation and projection
XGBoost: eXtreme Gradient Boosting
