# Supplementary material for: Cell-specific multi-target mechanisms of cotinine in cervical cancer oncogenesis and progression
Source: Discov Oncol. 2026 Jun 12;17:1204. doi: 10.1007/s12672-026-05375-5 (PMC13486414; doi:10.1007/s12672-026-05375-5)
Supplement: Supplementary file 1 [file 12672_2026_5375_MOESM1_ESM.docx]

Figure S1.Molecular docking and molecular dynamics simulation analysis of cotinine binding to *CA9*. **A** Downregulated genes were identified from the 17 key targets obtained by machine learning based on transcriptomic analysis; **B** Molecular docking results; **C-D** Root-mean-square deviation (RMSD) curves of protein-ligand complexes; **E** Root-mean-square fluctuation (RMSF) analysis at the protein residue level; **F** Radius of gyration curves of protein-ligand complexes; **G** Protein conformational adjustment and its interaction with solvent; **H** Dynamic changes in the number of hydrogen bonds between protein and ligand; **I** Covariance matrix heatmaps of protein residue movements; **J** Two-dimensional Gibbs free energy landscape based on principal component analysis; **K** Three-dimensional Gibbs free energy landscape based on principal component analysis.

Figure S2. Molecular docking and molecular dynamics simulation analysis of cotinine binding to *CCNB1*. **A** Downregulated genes were identified from the 17 key targets obtained by machine learning based on transcriptomic analysis; **B** Molecular docking results; **C-D** Root-mean-square deviation (RMSD) curves of protein-ligand complexes; **E** Root-mean-square fluctuation (RMSF) analysis at the protein residue level; **F** Radius of gyration curves of protein-ligand complexes; **G** Protein conformational adjustment and its interaction with solvent; **H** Dynamic changes in the number of hydrogen bonds between protein and ligand; **I** Covariance matrix heatmaps of protein residue movements; **J** Two-dimensional Gibbs free energy landscape based on principal component analysis; **K** Three-dimensional Gibbs free energy landscape based on principal component analysis.

Figure S3. In Silico Knockout Analysis of *CA9*. **A** Barplot of the top 20 differentially regulated genes; **B-D** Barplots of GO, KEGG and Reactome enrichment analyses for stable knockout-responsive genes.

Figure S4. In Silico Knockout Analysis of *CCNB1*. **A** Barplot of the top 20 differentially regulated genes; **B-D** Barplots of GO, KEGG and Reactome enrichment analyses for stable knockout-responsive genes.
